# The Effect of Integrated Care Management on Dementia in Atrial Fibrillation

**DOI:** 10.3390/jcm9061696

**Published:** 2020-06-02

**Authors:** Pil-Sung Yang, Jung-Hoon Sung, Eunsun Jang, Hee Tae Yu, Tae-Hoon Kim, Jae-Sun Uhm, Jong-Youn Kim, Hui-Nam Pak, Moon-Hyoung Lee, Gregory Y. H. Lip, Boyoung Joung

**Affiliations:** 1 Department of Cardiology, CHA Bundang Medical Centre, CHA University, Seongnam 13496, Korea; psyang01@cha.ac.kr (P.-S.Y.); atropin5@cha.ac.kr (J.-H.S.); 2 Division of Cardiology, Department of Internal Medicine, Severance Cardiovascular Hospital, Yonsei University College of Medicine, Seoul 03722, Korea; SUNNY_JES@yuhs.ac (E.J.); HEETYU@yuhs.ac (H.T.Y.); THKIMCARDIO@yuhs.ac (T.-H.K.); JASON@yuhs.ac (J.-S.U.); jykim0706@yuhs.ac (J.-Y.K.); HNPAK@yuhs.ac (H.-N.P.); MHLEE@yuhs.ac (M.-H.L.); 3 Liverpool Centre for Cardiovascular Science, University of Liverpool and Liverpool Heart & Chest Hospital, Liverpool L14 3PE, UK

**Keywords:** atrial fibrillation, dementia, integrated, Alzheimer

## Abstract

Clinical outcomes of patients with atrial fibrillation (AF) can be improved by an integrated care approach. We analyzed whether adherence with the AF Better Care (ABC) pathway for integrated care management would reduce the risk of dementia in a nationwide AF cohort. Using the National Health Insurance Service database of Korea, 228,026 non-valvular AF patients were retrospectively evaluated between 2005 and 2015. Patients meeting all criteria of the ABC pathway were classified as the “ABC” group and those not classified as the “non-ABC” group. During a median (25th, 75th percentiles) follow-up of 6.0 (3.3, 9.5) years, the ABC group had lower rates and risk of overall dementia (0.17 vs. 1.11 per 100 person-years, *p* < 0.001; hazard ratio (HR) 0.80; 95% CI 0.73–0.87) and both Alzheimer’s (HR 0.79, 95% CI 0.71–0.88) and vascular dementia (HR 0.76, 95% CI 0.59–0.98) than the non-ABC group. The stratified analysis showed that the ABC pathway reduced the risk of dementia regardless of sex, comorbidities, and in patients with high stroke risk. Adherence with the ABC pathway is associated with a reduced risk of dementia in AF patients. Due to the high medical burden of AF, it is necessary to implement integrated AF management to reduce the risk of dementia.

## 1. Introduction

Atrial fibrillation (AF) is the most common cardiac arrhythmia and is therefore a substantial economic and public health burden [1,2]. The age distribution of AF is predicted to shift with an expected increase in prevalence among the elderly [2]. AF increases the risk of mortality and morbidity with a concurrent increase in associated conditions including stroke, congestive heart failure, and hospitalization. AF also increases the occurrence of comorbid chronic diseases [3]. Little is known about the pathophysiological mechanisms of dementia, but there is increasing evidence that AF is related to cognitive dysfunction and dementia [4,5,6,7].

Recent trials involving AF revealed a high (4.6% per year) rate of all-cause death in patients with AF, with only 10% of deaths related to stroke and approximately 50%–60% deaths related to cardiovascular causes [8,9]. Therefore, recent AF guidelines advocated a more integrated approach beyond anticoagulation therapy for patients with AF to reduce death and adverse outcomes in AF [10,11,12]. The ABC (atrial fibrillation Better Care) pathway has been proposed as such a simple and holistic integrated approach [13]. This pathway facilitates the care pathway as follows: “A”—avoid stroke with optimal anticoagulation; “B”—better symptom management; and “C”—cardiovascular and comorbidity management [13]. The application of the simple ABC pathway was associated with a lower risk of all-cause death and the composite outcome included all-cause death, ischemic stroke, myocardial infarction, or major bleeding in patients with AF [14,15,16]. However, population-based benefits for total dementia, Alzheimer’s, or vascular dementia due to adherence to the ABC pathway in AF pathway patients have not been previously assessed in AF patients. Given the close association between AF and dementia, this study aimed to evaluate whether compliance with the ABC pathway would improve the risk of dementia in patients with AF.

## 2. Experimental Section

All data and materials have been made publicly available at the National Health Insurance Service (NHIS) of Korea. The data can be accessed on the National Health Insurance Data Sharing Service homepage of the NHIS (http://nhiss.nhis.or.kr). This study was a retrospective cohort analysis using the national health claims database (NHIS-2016-4-009) established by the NHIS of Korea [1,2,17]. The NHIS is the single insurer managed by the Korean government, and the majority (97.1%) of the Korean population are mandatory subscribers, with the remaining 3% of the population being medical aid subjects. The NHIS claims database includes diagnoses, biochemical test results, prescription records, procedures, and demographic information. This study was approved by the Institutional Review Board of Yonsei University Health System (4-2016-0179). 

### 2.1. Study Cohort 

**Figure 1:**
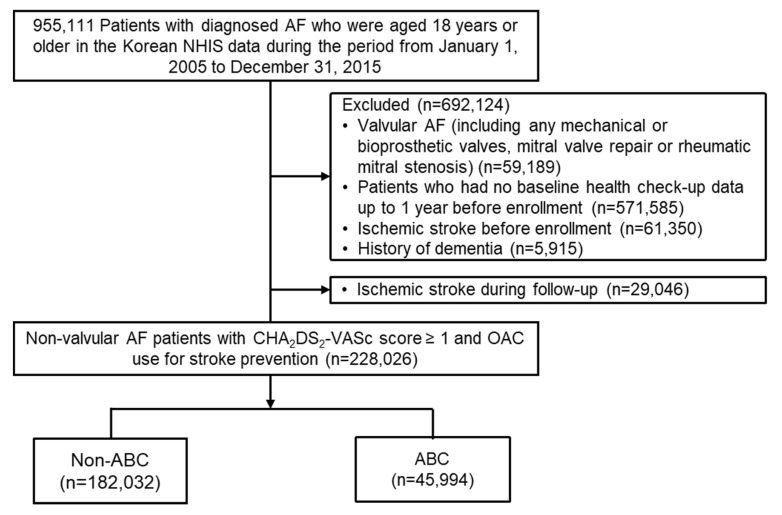
Flowchart of study population enrolment. ABC, atrial fibrillation better care; AF, atrial fibrillation; OAC, oral anticoagulant; NHIS, National Health Insurance Service.

In the Korean NHIS database, a total of 955,111 patients with prevalent AF over 18 years of age were identified from January 1, 2005 to December 31, 2015. Patients with valvular AF, such as those with rheumatic mitral stenosis, or surgery of heart valves (*n* = 59,189), those without baseline health check-up data up to 1 year before enrolment (*n* = 571,585), those who had ischemic stroke (*n* = 61,350), and those with history of dementia (*n* = 5915) were excluded. Patients with ischemic stroke during the follow-up period were additionally excluded (*n* = 29,046). Finally, a total of 228,026 patients with non-valvular AF were enrolled in the study to evaluate the effect of the ABC pathway on the development of dementia (Figure 1). 

AF was diagnosed using the International Classification of Disease 10th revision code I48. To ensure diagnostic accuracy, AF was defined as being present only if it was a discharge diagnosis or confirmed more than once in the outpatient department. The diagnosis of AF was previously verified in the NHIS database with a positive predictive value of 94.1% [2,18,19,20].

### 2.2. Definition of the ABC Pathway-Compliant Group

The ABC pathway was defined according to the criteria outlined in Supplementary Figure S1. “A” was defined as stroke prevention in accordance with the guidelines [12]. The patients with optimal anticoagulation included those with a high adherence of oral anticoagulants with a prescription covering ≥80% of days; “B” was defined in relation to visits requiring medical contact with an outpatient clinic (less than 5 visits per year during the tracking period); “C” was defined as optimal management of comorbidities and obesity. Optimal management of hypertension was defined as baseline systolic blood pressure values less than 140 mmHg and diastolic blood pressure values less than 90 mmHg. For other comorbidities including heart failure, myocardial infarction, peripheral artery disease, stroke/transient ischemic attack (TIA), and diabetes mellitus, the appropriate use of cardiovascular medications according to current guidelines was considered optimal management. For obesity, a body mass index less than 30 kg/m^2^ was considered optimal management. Patients meeting all criteria were classified as the “ABC” group and those not classified as the “non-ABC” group. 

### 2.3. Covariates, Primary and Secondary Outcome

Information regarding the comorbidity conditions was obtained from inpatient and outpatient hospital diagnoses. Baseline comorbidities were defined using medical claims and prescription medications before the index date. Similar to previous studies using NHIS data, the patients were considered to have comorbidities when the condition was a discharge diagnosis or was confirmed at least twice in an outpatient setting (Supplementary Table S1) [1,2,7,17,18,21]. 

The primary outcome was the initial occurrence of overall dementia. Secondary outcomes included the development of dementia subtypes, including Alzheimer’s disease and vascular dementia. Medical expenditure for dementia patients is covered by the Korean government. For the diagnosis of dementia, we used the following ICD-10 codes of dementia (F00 or G30 for Alzheimer’s disease, F01 for vascular dementia, F02 for dementia with other diseases classified elsewhere, and F03 or G31 for unspecified dementia) and dementia drugs (rivastigmine, galantamine, memantine, or donepezil). To access the accuracy of the dementia definitions, validation studies were conducted in two teaching hospitals with a total of 972 patients using patient medical records and the results of cognitive function tests. The positive predictive value was 94.7% [7]. 

Patients were followed up from the index date until the date of occurrence of the study outcomes, or until the end of follow-up, whichever came first. Each endpoint was analyzed independently from the others. 

### 2.4. Statistical Analysis

Categorical data are reported as proportions, while continuous data are reported as medians with 25th and 75th percentiles. Baseline characteristics between outcomes between the ABC (i.e., integrated care) and non-ABC groups were compared using Student’s *t*-test and Pearson’s chi-square test. Event rates were defined as events per 100 person-years at risk but expressed as annualized percentage rates for comprehensiveness. The relationships between the total number of ABC criteria fulfilled and the clinical outcomes were also investigated. Cox proportional hazards regressions were used to compare adverse outcomes between the ABC and non-ABC groups. Clinical variables including age, sex, economic status, heart failure, hypertension, diabetes, myocardial infarction, peripheral arterial disease CHA_2_DS_2_-VASc score, and modified HAS-BLED score were adjusted. *p*-values < 0.05 were considered significant. Statistical analyses were conducted using SAS version 9.3 (SAS Institute, Cary, NC, USA) and *R* version 3.3.2 (The *R* Foundation, www.R-project.org).

## 3. Results

### 3.1. Baseline Characteristics

Comparisons between the ABC and non-ABC groups are presented in Table 1. Compared with the non-ABC group, patients in the ABC group had a lower median age (49 vs. 64 years, *p* < 0.001), and a lower prevalence of comorbidities. In addition, patients in the ABC group had lower mean CHA_2_DS_2_-VASc and modified HAS-BLED scores than that of patients in the non-ABC group.

The multivariable analysis showed that female, high economic status, chronic kidney disease, chronic obstructive pulmonary disease (COPD), and history of malignancy were related to compliance with the ABC pathway (Table 2). However, older age, heart failure, hypertension, and other comorbidities were related to non-compliance with the ABC pathway.

### 3.2. Dementia

**Figure 2:**
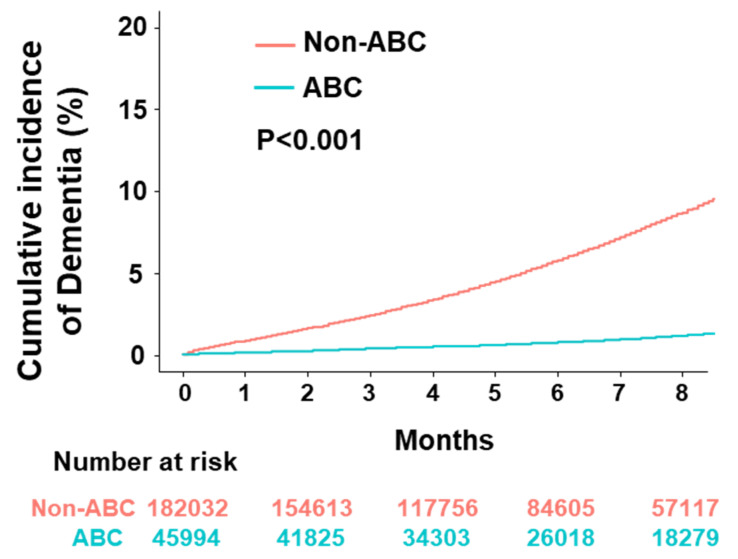
Cumulative incidences of dementia according to the use of integrated care (ABC).

During a median (25th, 75th percentiles) follow-up of 6.0 (3.3, 9.5) years, patients compliant with the ABC pathway had significantly lower cumulative incidences of dementia than those not compliant with the ABC pathway (Figure 2, Log rank *p*-value < 0.001). 

Compared with the non-ABC group, the ABC group had lower rates of dementia (0.17 vs. 1.11 per 100 person-years, *p* < 0.001). The adjustment of the clinical variables showed that ABC-compliant patients were associated with a 20% lower risk of dementia than non-ABC-compliant patients (hazard ratio (HR) 0.80, 95% confidence interval (CI) 0.73–0.87) (Table 3). Factors associated with the increased risk of dementia were older age (per 10 year increase, HR 3.28, 95% CI 3.15–3.43, *p* < 0.001), diabetes mellitus (HR 1.23, 95% CI 1.15–1.32, *p* < 0.001), and a higher CHA_2_DS_2_-VASc (per 1 score increase, HR 1.10, 95% CI 1.04-1.15, *p* < 0.001) or HAS-BLED score (per 1 score increase, HR 1.07, 95% CI 1.05–1.10, *p* < 0.001).

### 3.3. Alzheimer’s and Vascular Dementia 

**Figure 3:**
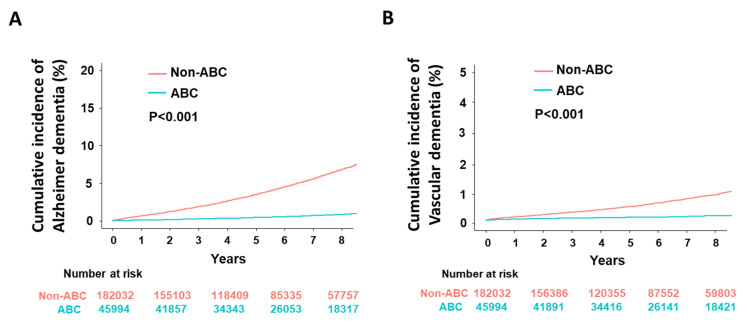
Cumulative incidences according to the use of integrated care (ABC). (**A**) Alzheimer’s dementia. (**B**) Vascular dementia.

Patients following the ABC pathway had significantly lower cumulative incidences of both Alzheimer’s and vascular dementia than those not following the ABC pathway (Figure 3, Log rank *p*-value < 0.001). 

Compared with the non-ABC group, the ABC group had lower rates of Alzheimer’s dementia (0.13 vs. 0.88 per 100 person-years, *p* < 0.001) and vascular dementia (0.02 vs. 0.11 per 100 person-years, *p* < 0.001). ABC-compliant patients were associated with a 21% lower risk of Alzheimer’s dementia (adjusted HR 0.79, 95% CI 0.71–0.88) and 24% lower risk of vascular dementia (adjusted HR 0.76, 95% CI 0.59–0.98) than non-ABC-compliant patients (Table 3).

### 3.4. Subgroup Analysis 

**Figure 4:**
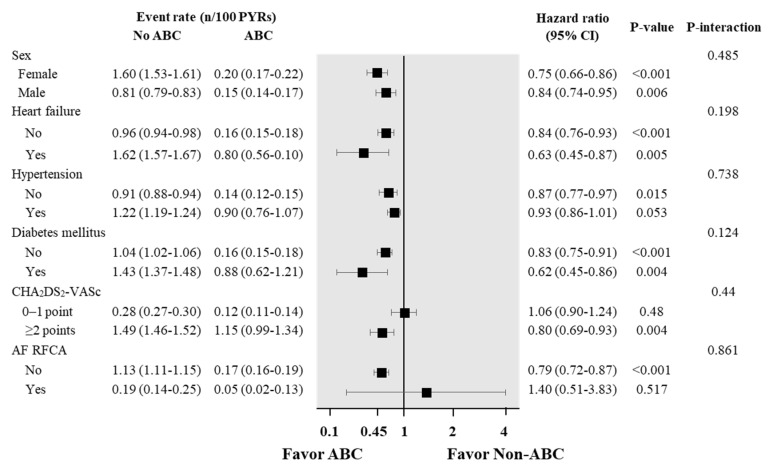
Events, event rates, and risk of dementia according to the use of integrated care (ABC) in different subgroups. CI = confidence interval; PYRs = person-years.

The stratified analysis showed that the ABC pathway reduced the risk of dementia regardless of sex, heart failure, hypertension, diabetes, and in patients with high stroke risk. AF ablation was performed in 4293 (2.4%) and 1318 (3.0%) in the non-ABC and ABC groups, respectively. The ABC pathway was related to a lower risk of dementia only in those without catheter ablation of AF (Figure 4). 

The risk of dementia for the non-ABC vs. ABC groups in different age subgroups is presented in Table 4. With the full adjustment, the ABC group had a significantly lower risk of dementia than the non-ABC group in elderly subjects (≥ 70 years; HR 0.82, 95%CI 0.69–0.98). With the age and sex adjustment, a lower risk was evident for age ≥60 (HR 0.86; 95%CI 0.75–0.99).

## 4. Discussion

In this largest nationwide population-based study, our principal findings were as follows: (i) the event rates and risks of overall dementia were all significantly lower in the ABC group than in the non-ABC group; (ii) compared with patients in the non-ABC group, those in the ABC group had a lower risk of Alzheimer’s or vascular dementia; and (iii) the ABC group was associated with the reduced risk of dementia regardless of sex, comorbidities, and in patients with high stroke risk. Given the high healthcare burden associated with AF, a streamlined holistic approach to the management of AF would improve dementia in such patients.

### 4.1. AF and Dementia 

There are approximately 40 million people living with dementia worldwide, and this number is expected to increase with a rising aged population [22]. Little is known about the pathophysiological mechanisms of dementia, but evidence is accumulating that AF may contribute to the development of cognitive dysfunction and dementia [4,5]. According to the Rotterdam Study, cognitive dysfunction was about two times more common in subjects with AF than in those without AF [4]. Since then, several longitudinal studies have shown that AF is independently associated with an increased risk of cognitive decline or dementia [5,7]. Kim et al. [7]. also found that the risk of dementia was increased by 65% in patients with AF than those without AF, even after censoring for incident stroke. In addition, the risk of both Alzheimer’s and vascular dementia was increased by incident AF.

### 4.2. Dementia and Integrated AF Management 

The use of an integrated care approach to AF management has been associated with reduced cardiovascular hospitalisation and all-cause mortality [23]. The ABC pathway was proposed to streamline interventions and decision-making, and to optimize the patient management pathway, providing simple guidance for the key components of integrated care [13]. Recent AF management guidelines have incorporated the ABC pathway [12,24]. Yoon et al. [16] showed that the following the ABC pathway was associated with improved clinical outcomes in patients with AF. Compliance to the ABC pathway was also associated with a lower risk of stroke, heart failure, and acute MI, as well as major bleeding, in patients with AF [16].

In this study, the ABC group showed a reduced risk of overall and both Alzheimer’s or vascular dementia in patients with AF. In the age subgroup analysis, with the full adjustment, the ABC group had a significantly lower risk of dementia than the non-ABC group in elderly subjects (≥70 years). With the age and sex adjustment, a lower risk was evident for age ≥60, with point estimates for age <60 suggestive of a modest benefit with ABC, but limited by small numbers and low event rates.

The reduction in dementia in the ABC group might be related to the adherence of oral anticoagulation and the control of risk factors. We and other groups demonstrated that the use of oral anticoagulants was linked with a decreased incidence of dementia, especially in the senior population [7,25]. Ischemic stroke is closely related with the risk of dementia [7]. The adherent use of NOAC showed a lower risk of ischemic stroke, which increases the risk of dementia, without increasing the bleeding risk [26]. The decreased risk of ischemic stroke was observed in AF patients with a strict control of blood pressure [18,21] or the glucose level [27]. A recent study showed that the strict control of blood pressure was also related to the reduction in dementia [28]. 

The strong impact of the ABC pathway on dementia substantiates and strengthens the concept that a holistic approach for integrated management is associated with a significant clinical benefit for patients with AF. 

### 4.3. Study Limitations

The present study has some limitations. Although administrative databases are increasingly used for clinical studies, such researches can lead to inaccurate results due to coding errors. To minimize this problem, we applied the definition that we had validated in previous studies using the Korean NHIS database [1,2,7,17,18,21]. Second, we used a high adherence of oral anticoagulants with a prescription covering ≥80% of days as a surrogate of optimal anticoagulation. However, we could not evaluate the TTR data in the warfarin patients, or label adherence prescribing of non-vitamin K oral anticoagulants. Third, this study defined the low frequency of medical contact as a better symptom of management. However, a symptom is just one of the factors to decide the frequency of medical contact. Despite these limitations, to the best of our knowledge, this study presents the largest nationwide population dataset available in the literature to investigate the relationship between integrated management and dementia in patients with AF.

## 5. Conclusions

Compliance with the simple ABC pathway is associated with reduced dementia in patients with AF. Due to the high healthcare burden associated with AF, such a streamlined holistic approach to the management of AF should be implemented in order to reduce dementia in AF patients. 

## Figures and Tables

**Table 1:** Comparison of clinical characteristics between atrial fibrillation patients compliant with and without the atrial fibrillation Better Care (ABC) pathway.

	Non-ABC (*n* = 182,032)	ABC (*n* = 45,994)	*p*-Value
Age, years	64 (55–71)	49 (41–57)	<0.001
Female	70,218 (38.6)	18,016 (39.2)	0.019
Economic status	12 (5,17)	13 (6,17)	<0.001
CHA_2_DS_2_-VASc score	2 (1, 3)	0 (0, 1)	<0.001
mHAS-BLED score *	2 (1, 3)	0 (0, 1)	<0.001
Charlson comorbidity index	3 (1, 4)	1 (0, 2)	<0.001
Heart failure	41,557 (22.8)	627 (1.4)	<0.001
Hypertension	117,688 (64.7)	2425 (5.3)	<0.001
Diabetes	35,819 (19.7)	807 (1.8)	<0.001
Myocardial infarction	12,838 (7.1)	157 (0.3)	<0.001
Vascular disease	27,489 (15.1)	383 (0.8)	<0.001
Chronic kidney disease	6089 (3.3)	358 (0.8)	<0.001
Liver disease	70,974 (39.0)	13,365 (29.1)	<0.001
Malignancy	36,768 (20.2)	6876 (14.9)	<0.001
Hyperthyroidism	16,121 (8.9)	3498 (7.6)	<0.001
Hypothyroidism	13,870 (7.6)	2629 (5.7)	<0.001
COPD	23,033 (12.7)	1735 (3.8)	<0.001
History of bleeding	12,525 (6.9)	1667 (3.6)	<0.001
Medication			
Oral anticoagulants (Baseline)	5822 (3.2)	1166 (2.5)	<0.001
Oral anticoagulants (Follow-up)	46,569 (25.6)	7705 (16.8)	<0.001
Antiplatelet agents	68,141 (37.4)	2339 (5.1)	<0.001
Beta blocker	59,148 (32.5)	2794 (6.1)	<0.001
Statin	44,226 (24.3)	2791 (6.1)	<0.001
Calcium channel blocker	69,697 (38.3)	1664 (3.6)	<0.001
ACE-inhibitor/ARB	66,533 (36.6)	1605 (3.5)	<0.001
Antiarrhythmic drugs ^†^	7087 (3.9)	544 (1.2)	<0.001

ABC = atrial fibrillation Better Care, ACE = angiotensin converting enzyme, AF = atrial fibrillation, ARB = angiotensin II receptor blocker, COPD = chronic obstructive pulmonary disease. * Modified HASBLED = hypertension, 1 point; >65 years old, 1 point; stroke history, 1 point; bleeding history or predisposition, 1 point; liable international normalized ratio, not assessed; ethanol or drug abuse, 1 point; drug predisposing to bleeding, 1 point. ^†^ Antiarrhythmic drugs included class Ic arrhythmic drugs (e.g., flecainide, propafenone, pilsicainide), and class III drugs (e.g., sotalol, dronedarone, amiodarone). Values are presented as numbers (%) or median (Q1, Q3, quartiles (25th and 75th percentiles)).

**Table 2:** Factors associated with the ABC pathway.

	Multivariable Adjustment	
	OR (95% CI)	*p*-Value
Age (per 10-year increase)	0.91 (0.90–0.91)	<0.001
Female	1.05 (1.05–1.05)	<0.001
High economic status	1.02 (1.01–1.02)	<0.001
Heart failure	0.97 (0.96–0.97)	<0.001
Hypertension	0.76 (0.76–0.76)	<0.001
Diabetes	0.93 (0.92–0.93)	<0.001
Myocardial infarction	0.96 (0.95–0.96)	<0.001
Peripheral arterial disease	0.97 (0.96–0.97)	<0.001
Chronic kidney disease	1.02 (1.01–1.03)	<0.001
COPD	1.02 (1.01–1.02)	<0.001
Liver disease	0.98 (0.98–0.98)	<0.001
Malignancy	1.02 (1.01–1.02)	<0.001
Hyperthyroidism	0.98 (0.98–0.99)	<0.001
Hypothyroidism	0.99 (0.99–1.00)	0.011
Intracranial hemorrhage	0.97 (0.95–0.98)	<0.001

COPD = chronic obstructive pulmonary disease.

**Table 3:** Dementia, Alzheimer’s, and vascular dementia hazard ratios for the non-ABC vs. ABC groups.

	Cases, *n* (%)	Event Rate (/100 Person-Years)	Age & Sex Adjusted HR (95% CI)	Adjusted HR (95% CI) *
Dementia				
Non-ABC	12,165	1.11 (1.09–1.13)	1 (Reference)	1 (Reference)
ABC	538	0.17 (0.16–0.19)	0.74 (0.68–0.81)	0.80 (0.73–0.87)
Alzheimer’s dementia				
Non-ABC	9731	0.88 (0.86–0.90)	1 (Reference)	1 (Reference)
ABC	410	0.13 (0.12–0.14)	0.74 (0.67–0.82)	0.79 (0.71–0.88)
Vascular dementia				
Non-ABC	1274	0.11 (0.11–0.12)	1 (Reference)	1 (Reference)
ABC	72	0.02 (0.18–0.29)	0.68 (0.53–0.88)	0.76 (0.59–0.98)

CI = confidence interval, HR = hazard ratio. * Clinical variables including age, sex, economic status, heart failure, hypertension, diabetes, myocardial infarction, peripheral arterial disease CHA2DS2-VASc score, and modified HAS-BLED score were adjusted.

**Table 4:** Risk of dementia for the non-ABC vs. ABC groups in different age subgroups.

	Cases, *n* (%)	Event Rate (/100 Person-Years)	Age & Sex Adjusted HR (95% CI)	Adjusted HR (95% CI) *
≥70 years (*n* = 57,480)				
Non-ABC	8186	3.05 (2.98–3.11)	1 (Reference)	1 (Reference)
ABC	129	2.22 (1.86–2.64)	0.83 (0.70–0.99)	0.82 (0.69–0.98)
≥60 and <70 years (*n* = 67,073)				
Non-ABC	3415	0.89 (0.86–0.92)	1 (Reference)	1 (Reference)
ABC	221	0.52 (0.45–0.59)	0.86 (0.75–0.99)	0.93 (0.81–1.08)
≥50 and <60 years (*n* = 56,161)				
Non-ABC	503	0.18 (0.17–0.20)	1 (Reference)	1 (Reference)
ABC	147	0.15 (0.13–0.18)	0.92 (0.77–1.11)	1.05 (0.84–1.30)
<50 years (*n* = 47,312)				
Non-ABC	61	0.03 (0.03–0.04)	1 (Reference)	1 (Reference)
ABC	41	0.02 (0.02–0.03)	0.86 (0.58–1.30)	0.94 (0.58–1.54)

CI = confidence interval, HR = hazard ratio. * Clinical variables including age, sex, economic status, heart failure, hypertension, diabetes, myocardial infarction, peripheral arterial disease CHA2DS2-VASc score, and modified HAS-BLED score were adjusted.

## Supplementary Materials

The following are available online at https://www.mdpi.com/2077-0383/9/6/1696/s1, Supplementary Table S1: Definitions and ICD-10 codes used for defining the comorbidities and clinical outcomes. Supplementary Table S2: Comparison of baseline characteristics between atrial fibrillation patients compliant with and without ABC pathway. Supplementary Figure S1. The ABC pathway for integrated care management.
