# Supplementary material for: The Effect of Integrated Care Management on Dementia in Atrial Fibrillation
Source: J Clin Med. 2020 Jun 2;9(6):1696. doi: 10.3390/jcm9061696 (PMC7356978; doi:10.3390/jcm9061696)
Supplement: Supplementary file 1 [file jcm-09-01696-s001.pdf]

Supplementary Materials

Supplementary Table 1. Definitions and ICD-10 codes used for defining the comorbidities and clinical outcomes.

	**Definitions**	**ICD-10 codes or conditions**
Atrial fibrillation[1]	Defined from diagnosis*	I48
Heart failure[2]	Defined from diagnosis*	ICD-10: I11.0, I50, I97.1
Diabetes mellitus[2]	Defined from diagnosis* plus treatment	ICD-10: E10, E11, E12, E13, E14<br>Treatment: all kinds of oral antidiabetics and insulin.
Myocardial infarction[3]	Defined from diagnosis*	ICD-10: I21, I22, I25.2
Peripheral arterial disease[2]	Defined from diagnosis*	ICD-10: I70.0, I70.1, I70.2, I70.8, I70.9
Chronic kidney disease[2]	Defined from eGFR or diagnosis*<br>(if laboratory value was not available, diagnosis code was used)	eGFR <60mL/min per 1.73 m^2<br>ICD-10: N18, N19
Dyslipidemia[2]	Defined from diagnosis*	ICD-10: E78
Hypertension[2]	Defined from diagnosis*	I10, I11, I12, I13, I15 and antihypertensive medication
TIA[2]	Defined from diagnosis*	G45
Arterial embolism[2]	Defined from diagnosis*	I74, N280
Chronic Liver disease	Defined from diagnosis of chronic liver disease, cirrhosis, and hepatitis	B18, K70, K71, K72, K73, K74, K76.1
Chronic obstructive pulmonary disease[4]	Defined from diagnosis* plus treatment	J42, J43(except J43.0), J44<br>Treatment: SABA, SAMA, LABA, LAMA, ICS, ICS+LABA, or methylxanthine (>1 months).
Malignancy	Defined from diagnoses of cancer (non-benign)	C00-C97
ESRD[5]	Defined from national registry for severe illness.	Patients with ESRD undergoing chronic dialysis or received a kidney transplant.
Ischemic stroke[1, 6]	Defined from any discharge diagnoses with concomitant imaging studies	ICD-10: I63, I64 with concomitant brain-imaging studies, including computed tomography or magnetic resonance imaging.
Intracranial hemorrhage[7]	Defined from any discharge diagnoses with concomitant imaging studies	ICD-10: I60, I61, I62 with concomitant brain-imaging studies, including computed tomography or magnetic resonance imaging.

Gastrointestinal bleeding[3]	Defined from admission diagnosis or related death	K25-28 (subcodes 0-2 and 4-6 only), K92.0, K92.1, K92.2, K62.5, I85.0, I98.3
Respiratory or urinary tract bleeding, etc.[3]	Defined from admission diagnosis or related death	N02, R31, R04, J942, K661, D62

*To ensure accuracy, comorbidities were established based on one inpatient or two outpatient records of ICD-10 codes in the database. †To avoid erroneous inclusion of the patients with non-cardiac arrest, we excluded the patient with sudden arrest diagnosis accompanied by respiratory arrest (R09.0, R09.2), gastrointestinal bleeding (I85.0, K25.0, K25.4, K26.0, K26.4, K27.0, K27.4, K92.0-K92.2), brain hemorrhage (I60.x-I62.x, S06.4-S06.6), septic shock (A41.9, R57.2), pregnancy and delivery (O00-O99), diabetic ketoacidosis (E14.1), anaphylaxis (T78.2), and accidents including suicide (T71, T75.1, T36-T65, V80-V89, W76.x, X60-X84). eGFR = estimated glomerular filtration rate; ICD-10 = International Classification of Diseases-10th Revision. All covariates were validated in cited references.

Supplementary Table 2. Comparison of baseline characteristics between atrial fibrillation patients compliant with and without ABC pathway**.**

	**≥ 70 years**			**≥ 60 and <70 years**			**≥ 50 and <60 years**			**<50 years**		
	**Non-ABC (n=56,223)**	**ABC (n=1,257)**	**P-value**	**Non-ABC (n=60,417)**	**ABC (n=6,656)**	**P-value**	**Non-ABC (n=41,363)**	**ABC (n=14,798)**	**P-value**	**Non-ABC (n=24,029)**	**ABC (n=23,283)**	**P-value**
Age, years	74 (72, 78)	74 (72, 77)	0.001	65 (62, 67)	62 (61, 64)	<0.001	55 (53, 57)	54 (52, 57)	<0.001	44 (39, 47)	41 (34, 45)	<0.001
Female	25514 (45.4)	450 (35.8)	<0.001	24187 (40)	2498 (37.5)	<0.001	14236 (34.4)	6095 (41.2)	<0.001	6281 (26.1)	8973 (38.5)	<0.001
Economic status	14 (6,18)	15 (9,18)	<0.001	12 (5,17)	12 (5,17)	0.05	12 (5,17)	13 (6,18)	<0.001	13 (6,17)	13 (7,17)	0.263
CHA_2DS_2-VASc score	3 (2, 4)	3 (2, 4)	<0.001	2 (1, 3)	1 (0, 1)	<0.001	1 (1, 2)	0 (0, 1)	<0.001	1 (0, 2)	0 (0, 1)	<0.001
mHAS-BLED score*	1 (0, 2)	0 (0, 1)	0.396	2 (1, 3)	0 (0, 1)	<0.001	1 (1, 2)	0 (0, 1)	<0.001	1 (0, 2)	0 (0, 1)	<0.001
Charlson comorbidity index	3 (2, 5)	3 (1, 5)	<0.001	3 (1, 4)	2 (1, 3)	<0.001	2 (1, 4)	1 (1, 2)	<0.001	2 (1, 3)	1 (0, 2)	<0.001
Heart failure	17810 (31.7)	215 (17.1)	<0.001	13639 (22.6)	231 (3.5)	<0.001	7159 (17.3)	137 (0.9)	<0.001	2949 (12.3)	44 (0.2)	<0.001

Hypertension	39939 (71.0)	886 (70.5)	0.693	40160 (66.5)	929 (14.0)	<0.001	25988 (62.8)	473 (3.2)	<0.001	11601 (48.3)	137 (0.6)	<0.001
Diabetes	12070 (21.5)	301 (23.9)	0.038	12739 (21.1)	315 (4.7)	<0.001	8086 (19.5)	153 (1.0)	<0.001	2924 (12.2)	38 (0.2)	<0.001
Myocardial infarction	4565 (8.1)	35 (2.8)	<0.001	4185 (6.9)	63 (0.9)	<0.001	2703 (6.5)	43 (0.3)	<0.001	1385 (5.8)	16 (0.1)	<0.001
Vascular disease	10128 (18.0)	131 (10.4)	<0.001	9433 (15.6)	140 (2.1)	<0.001	5590 (13.5)	81 (0.5)	<0.001	2338 (9.7)	31 (0.1)	<0.001
Chronic kidney disease	2400 (4.3)	38 (3.0)	0.036	1852 (3.1)	80 (1.2)	<0.001	1230 (3.0)	116 (0.8)	<0.001	607 (2.5)	124 (0.5)	<0.001
Liver disease	20460 (36.4)	437 (34.8)	0.248	24262 (40.2)	2189 (32.9)	<0.001	17391 (42.0)	4757 (32.1)	<0.001	8861 (36.9)	5982 (25.7)	<0.001
Malignancy	14502 (25.8)	313 (24.9)	0.494	12712 (21.0)	1376 (20.7)	0.495	6871 (16.6)	2567 (17.3)	0.041	2683 (11.2)	2620 (11.3)	0.775
Hyperthyroidism	3881 (6.9)	102 (8.1)	0.106	5179 (8.6)	369 (5.5)	<0.001	4242 (10.3)	1075 (7.3)	<0.001	2819 (11.7)	1952 (8.4)	<0.001
Hypothyroidism	4072 (7.2)	116 (9.2)	0.009	4746 (7.9)	389 (5.8)	<0.001	3364 (8.1)	929 (6.3)	<0.001	1688 (7.0)	1195 (5.1)	<0.001
COPD	12765 (22.7)	214 (17.0)	<0.001	7382 (12.2)	536 (8.1)	<0.001	2336 (5.6)	592 (4.0)	<0.001	550 (2.3)	393 (1.7)	<0.001
History of bleeding	4840 (8.6)	82 (6.5)	0.01	4050 (6.7)	304 (4.6)	<0.001	2424 (5.9)	575 (3.9)	<0.001	1211 (5.0)	706 (3.0)	<0.001
Medication												
OAC (Baseline)	1777 (3.2)	230 (18.3)	<0.001	2150 (3.6)	368 (5.5)	<0.001	1198 (2.9)	302 (2.0)	<0.001	697 (2.9)	266 (1.1)	<0.001
OAC (Follow-up)	15411 (27.4)	1254 (99.8)	<0.001	17390 (28.8)	2243 (33.7)	<0.001	9674 (23.4)	2324 (15.7)	<0.001	4094 (17.0)	1884 (8.1)	<0.001
Antiplatelet agents	24727 (44.0)	565 (44.9)	0.512	24948 (41.3)	773 (11.6)	<0.001	13922 (33.7)	669 (4.5)	<0.001	4544 (18.9)	332 (1.4)	<0.001
Beta blocker	19208 (34.2)	414 (32.9)	0.38	20516 (34.0)	677 (10.2)	<0.001	13218 (32.0)	756 (5.1)	<0.001	6206 (25.8)	947 (4.1)	<0.001
Statin	14212	404	<0.00	16448	797	<0.001	10207	1122 (7.6)	<0.00	3359	468 (2.0)	<0.001

	(25.3)	(32.1)	1	(27.2)	(12.0)		(24.7)		1	(14.0)		
CCB	25701 (45.7)	590 (46.9)	<0.001	24641 (40.8)	598 (9.0)	<0.001	14338 (34.7)	336 (2.3)	<0.001	5017 (20.9)	140 (0.6)	<0.001
ACE-inhibitor/ARB	23330 (41.5)	563 (44.8)	0.021	23099 (38.2)	613 (9.2)	<0.001	14515 (35.1)	324 (2.2)	<0.001	5589 (23.3)	105 (0.5)	<0.001
AAD†	1731 (3.1)	42 (3.3)	0.653	2597 (4.3)	129 (1.9)	<0.001	1763 (4.3)	189 (1.3)	<0.001	996 (4.1)	184 (0.8)	<0.001

AAD=antiarrhythmic drugs, ACE=angiotensin converting enzyme, AF=atrial fibrillation, ARB=angiotensin II receptor blocker, CCB=calcium channel blocker, COPD=Chronic obstructive pulmonary disease, OAC=oral anticoagulants. *Modified HASBLED=hypertension, 1 point; >65 years old, 1 point; stroke history, 1 point; bleeding history or predisposition, 1 point; liable international normalized ratio, not assessed; ethanol or drug abuse, 1 point; drug predisposing to bleeding, 1 point. †Antiarrhythmic drugs included class Ic arrhythmic drugs (eg. flecainide, profapenone, pilcicainide), and class III drugs (eg. sotalol, dronedarone, amiodarone). Values are presented as numbers (%) or median (Q1, Q3, quartiles [25th and 75th percentiles]).

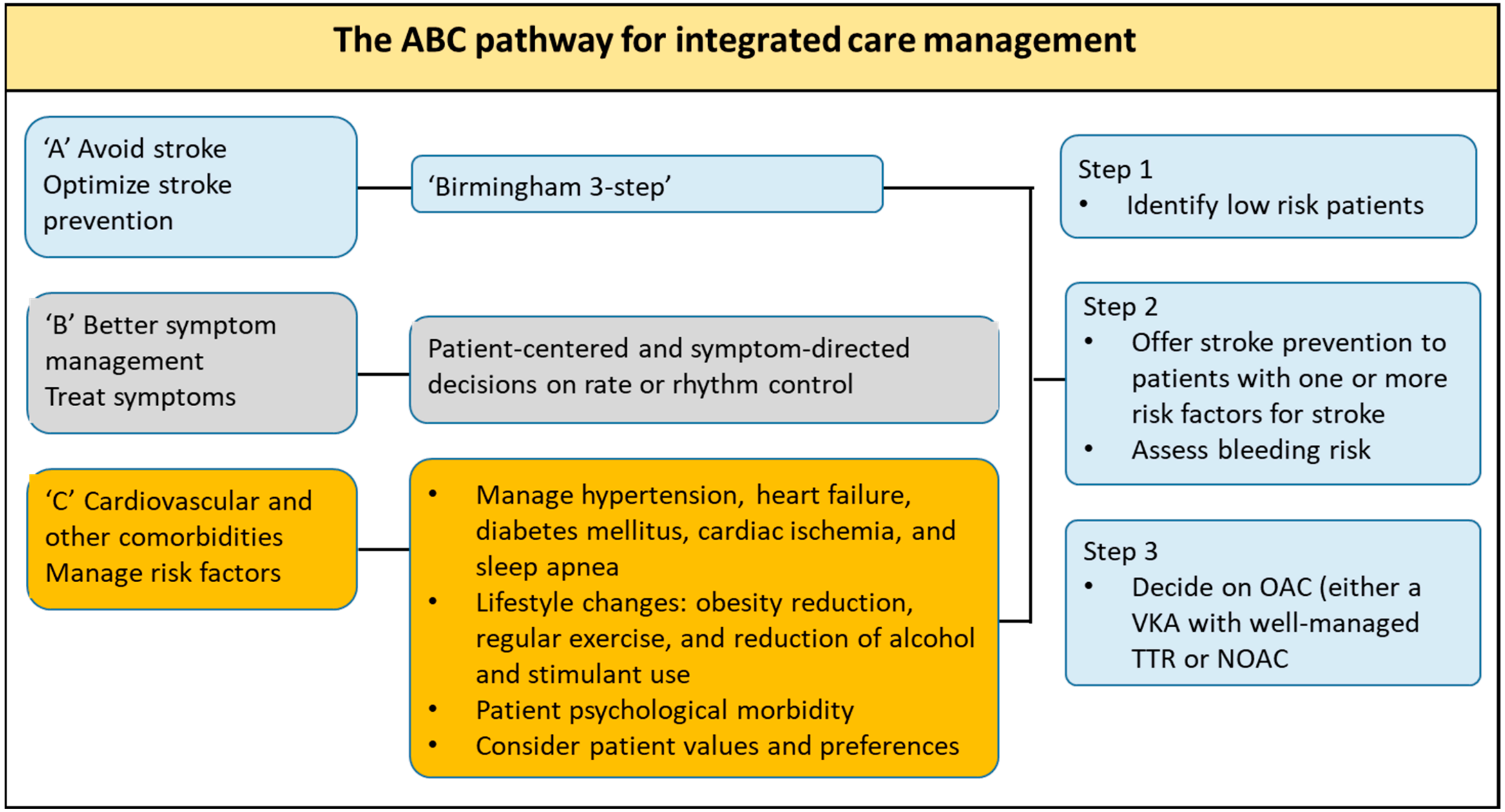

Supplementary Figure 1. The ABC pathway for integrated care management.
